# Supplementary material for: Unveiling the Bioactive Potential of the Invasive Jellyfish Phyllorhiza punctata Through Integrative Transcriptomic and Proteomic Analyses
Source: Biomolecules. 2025 Aug 4;15(8):1121. doi: 10.3390/biom15081121 (PMC12383608; doi:10.3390/biom15081121)
Supplement: Supplementary file 1 [file biomolecules-15-01121-s001.zip › Suplementary Material/FileS3/PEPFOLD4/XP_065058759_4648/clusters.html]

 PEP-FOLD clusters


Download archive of all models  here   

|  |  |  |
| --- | --- | --- |
| # | file | sOPEP |
| # cluster 1 - Size: 3 |  |  |
|  | XP\_065058759\_4648-model1 | -16.0977 |
|  | XP\_065058759\_4648-model1.1 | -15.4627 |
|  | XP\_065058759\_4648-model1.2 | -14.284 |
| # cluster 2 - Size: 61 |  |  |
|  | XP\_065058759\_4648-model2 | -11.2063 |
|  | XP\_065058759\_4648-model2.1 | -11.2063 |
|  | XP\_065058759\_4648-model2.2 | -11.2063 |
|  | XP\_065058759\_4648-model2.3 | -11.2063 |
|  | XP\_065058759\_4648-model2.4 | -11.2063 |
|  | XP\_065058759\_4648-model2.5 | -11.2063 |
|  | XP\_065058759\_4648-model2.6 | -11.1723 |
|  | XP\_065058759\_4648-model2.7 | -11.1723 |
|  | XP\_065058759\_4648-model2.8 | -11.1723 |
|  | XP\_065058759\_4648-model2.9 | -11.1304 |
|  | XP\_065058759\_4648-model2.10 | -11.0459 |
|  | XP\_065058759\_4648-model2.11 | -11.0459 |
|  | XP\_065058759\_4648-model2.12 | -11.0459 |
|  | XP\_065058759\_4648-model2.13 | -11.0459 |
|  | XP\_065058759\_4648-model2.14 | -11.0459 |
|  | XP\_065058759\_4648-model2.15 | -11.0459 |
|  | XP\_065058759\_4648-model2.16 | -11.0459 |
|  | XP\_065058759\_4648-model2.17 | -11.0459 |
|  | XP\_065058759\_4648-model2.18 | -11.006 |
|  | XP\_065058759\_4648-model2.19 | -10.9885 |
|  | XP\_065058759\_4648-model2.20 | -10.9885 |
|  | XP\_065058759\_4648-model2.21 | -10.9885 |
|  | XP\_065058759\_4648-model2.22 | -10.9885 |
|  | XP\_065058759\_4648-model2.23 | -10.9885 |
|  | XP\_065058759\_4648-model2.24 | -10.9448 |
|  | XP\_065058759\_4648-model2.25 | -10.9448 |
|  | XP\_065058759\_4648-model2.26 | -10.9448 |
|  | XP\_065058759\_4648-model2.27 | -10.9283 |
|  | XP\_065058759\_4648-model2.28 | -10.9283 |
|  | XP\_065058759\_4648-model2.29 | -10.9203 |
|  | XP\_065058759\_4648-model2.30 | -10.8564 |
|  | XP\_065058759\_4648-model2.31 | -10.8513 |
|  | XP\_065058759\_4648-model2.32 | -10.837 |
|  | XP\_065058759\_4648-model2.33 | -10.837 |
|  | XP\_065058759\_4648-model2.34 | -10.835 |
|  | XP\_065058759\_4648-model2.35 | -10.8287 |
|  | XP\_065058759\_4648-model2.36 | -10.8176 |
|  | XP\_065058759\_4648-model2.37 | -10.7872 |
|  | XP\_065058759\_4648-model2.38 | -10.7685 |
|  | XP\_065058759\_4648-model2.39 | -10.7639 |
|  | XP\_065058759\_4648-model2.40 | -10.76 |
|  | XP\_065058759\_4648-model2.41 | -10.7527 |
|  | XP\_065058759\_4648-model2.42 | -10.7527 |
|  | XP\_065058759\_4648-model2.43 | -10.7087 |
|  | XP\_065058759\_4648-model2.44 | -10.7087 |
|  | XP\_065058759\_4648-model2.45 | -10.7086 |
|  | XP\_065058759\_4648-model2.46 | -10.694 |
|  | XP\_065058759\_4648-model2.47 | -10.6741 |
|  | XP\_065058759\_4648-model2.48 | -10.6741 |
|  | XP\_065058759\_4648-model2.49 | -10.6323 |
|  | XP\_065058759\_4648-model2.50 | -10.6091 |
|  | XP\_065058759\_4648-model2.51 | -10.5921 |
|  | XP\_065058759\_4648-model2.52 | -10.5291 |
|  | XP\_065058759\_4648-model2.53 | -10.437 |
|  | XP\_065058759\_4648-model2.54 | -10.4067 |
|  | XP\_065058759\_4648-model2.55 | -10.3485 |
|  | XP\_065058759\_4648-model2.56 | -10.3362 |
|  | XP\_065058759\_4648-model2.57 | -10.247 |
|  | XP\_065058759\_4648-model2.58 | -10.2423 |
|  | XP\_065058759\_4648-model2.59 | -10.2401 |
|  | XP\_065058759\_4648-model2.60 | -10.1968 |
| # cluster 3 - Size: 6 |  |  |
|  | XP\_065058759\_4648-model3 | -11.0295 |
|  | XP\_065058759\_4648-model3.1 | -11.0203 |
|  | XP\_065058759\_4648-model3.2 | -10.9128 |
|  | XP\_065058759\_4648-model3.3 | -10.9104 |
|  | XP\_065058759\_4648-model3.4 | -10.8309 |
|  | XP\_065058759\_4648-model3.5 | -10.4615 |
| # cluster 4 - Size: 8 |  |  |
|  | XP\_065058759\_4648-model4 | -10.8641 |
|  | XP\_065058759\_4648-model4.1 | -10.8641 |
|  | XP\_065058759\_4648-model4.2 | -10.754 |
|  | XP\_065058759\_4648-model4.3 | -10.7157 |
|  | XP\_065058759\_4648-model4.4 | -10.6611 |
|  | XP\_065058759\_4648-model4.5 | -10.6611 |
|  | XP\_065058759\_4648-model4.6 | -10.4392 |
|  | XP\_065058759\_4648-model4.7 | -10.3647 |
| # cluster 5 - Size: 5 |  |  |
|  | XP\_065058759\_4648-model5 | -10.7711 |
|  | XP\_065058759\_4648-model5.1 | -10.5134 |
|  | XP\_065058759\_4648-model5.2 | -10.2626 |
|  | XP\_065058759\_4648-model5.3 | -10.1968 |
|  | XP\_065058759\_4648-model5.4 | -10.1161 |
| # cluster 6 - Size: 2 |  |  |
|  | XP\_065058759\_4648-model6 | -10.6959 |
|  | XP\_065058759\_4648-model6.1 | -10.3283 |
| # cluster 7 - Size: 12 |  |  |
|  | XP\_065058759\_4648-model7 | -10.5677 |
|  | XP\_065058759\_4648-model7.1 | -10.5112 |
|  | XP\_065058759\_4648-model7.2 | -10.438 |
|  | XP\_065058759\_4648-model7.3 | -10.3181 |
|  | XP\_065058759\_4648-model7.4 | -10.2005 |
|  | XP\_065058759\_4648-model7.5 | -10.1793 |
|  | XP\_065058759\_4648-model7.6 | -10.1595 |
|  | XP\_065058759\_4648-model7.7 | -10.1456 |
|  | XP\_065058759\_4648-model7.8 | -10.1 |
|  | XP\_065058759\_4648-model7.9 | -10.0734 |
|  | XP\_065058759\_4648-model7.10 | -10.0466 |
|  | XP\_065058759\_4648-model7.11 | -10.0405 |
| # cluster 8 - Size: 2 |  |  |
|  | XP\_065058759\_4648-model8 | -10.4665 |
|  | XP\_065058759\_4648-model8.1 | -10.4337 |
| # cluster 9 - Size: 1 |  |  |
|  | XP\_065058759\_4648-model9 | -10.1476 |
|  |  |  |
